# Ultrasound-guided central venous catheterization in cancer patients improves the success rate of cannulation and reduces mechanical complications: A prospective observational study of 1,978 consecutive catheterizations

**DOI:** 10.1186/1477-7819-8-91

**Published:** 2010-10-19

**Authors:** Luigi Cavanna, Giuseppe Civardi, Daniele Vallisa, Camilla Di Nunzio, Lorella Cappucciati, Raffaella Bertè, Maria Rosa Cordani, Antonio Lazzaro, Gabriele Cremona, Claudia Biasini, Monica Muroni, Patrizia Mordenti, Silvia Gorgni, Elena Zaffignani, Massimo Ambroggi, Livia Bidin, Maria Angela Palladino, Carmelina Rodinò, Laura Tibaldi

**Affiliations:** 1 Oncology-Hematology Department, Hospital of Piacenza, Piacenza, Italy; 2 Department of Medicine, AUSL Piacenza, Italy; 3 Teaching and management Department of Nursing Staff, AUSL of Piacenza, Italy

## Abstract

**Background:**

A central venous catheter (CVC) currently represents the most frequently adopted intravenous line for patients undergoing infusional chemotherapy and/or high-dose chemotherapy with hematopoietic stem-cell transplantation and parenteral nutrition.

CVC insertion represents a risk for pneumothorax, nerve or arterial punctures. The aim of this prospective observational study was to explore the safety and efficacy of CVC insertion under ultrasound (US) guidance and to confirm its utility in clinical practice in cancer patients.

**Methods:**

Consecutive adult patients attending the oncology-hematology department were eligible if they had solid or hematologic malignancies and required CVC insertion. Four types of possible complication were defined a priore: mechanical, thrombotic, infection and malfunctioning.

The patient was placed in Trendelenburg's position, a 7.5 MHZ puncturing US probe was placed in the supraclavicular site and a 16-gauge needle was advanced under real-time US guidance into the last portion of internal jugular vein. The Seldinger technique was used to place the catheter, which was advanced into the superior vena cava until insertion into right atrium. Within two hours after each procedure, an upright chest X-ray and ultrasound scanning were carried out to confirm the CVC position and to rule out a pneumotorax. CVC-related infections, symptomatic vein thrombosis and malfunctioning were recorded.

**Results:**

From December 2000 to January 2009, 1,978 CVC insertional procedures were applied to 1,660 consecutive patients. The procedure was performed 580 times in patients with hematologic malignancies and 1,398 times those with solid tumors. A single-needle puncture of the vein was performed on 1,948 of 1,978 procedures (98.48%); only eighteen attempts among 1,978 failed (0.9%).

No pneumotorax, no major bleeding, and no nerve puncture were reported; four cases (0.2%) showed self-limiting hematomas. The mean lifespan of CVC was 189.7 +/- 18.6 days (range 7-701). Symptomatic deep-vein thrombosis of the upper limbs developed in 48 patients (2.42%). Catheter-related infections occurred in 197 (9.96%) of the catheters inserted. They were successfully treated with antibiotics and only in 48 (2.9%) patients definitive CVC removal was required for infection and/or thrombosis or malfunctioning.

**Conclusions:**

This study represents the largest published series of consecutive patients with cancer undergoing CVC insertion under US guidance; this procedure allowed the completion of the therapeutic program for 1,930/1,978 (97.6%) of the catheters inserted. The absence of pneumotorax and other major complications indicates that US guidance should be mandatory for CVC insertion in patients with cancer.

## Background

Central venous access is essential in patients with cancer, and the need for intravenous access devices for the administration of cancer therapy has increased proportionally with the increasing number of patients diagnosed with cancer. The percutaneous approach to the subclavian or internal jugular vein is currently the most popular procedure for placing catheters in the superior vena cava both for short-term and long-term use.

Unfortunately, central venous catheter (CVC) insertion represents a risk of pneumothorax, nerve puncture and major bleeding (mechanical complications), infection and CVC-related vein thrombosis [1,2].

Mechanical complications of CVC insertion without ultrasound (US) guidance, such as arterial puncture and pneumothorax, are seen in up to 21% of attempts, and up to 35% of insertion attempts are not successful [3-5].

There have been several prospective randomized trials [6-14] and two metanalyses [15,16] that suggest the use of US has been associated with a reduction in complication rate and an improved first-pass success when placing CVC in the internal jugular vein.

In 2001, the U.S. Agency for Healthcare Research and Quality recommended the use of ultrasound for the placement of CVC as one of their 11 practices to improve patient care [17,18]. However a survey of 250 anesthetists in the United Kingdom found that 41% disagreed or strongly disagreed with the recommendation that ultrasound imaging should be the preferred method for insertion of a central venous catheter in the internal jugular vein [19].

In addition, a report in the United States also showed that <15% of surgery, anesthesia, internal medicine, emergency medicine and family medicine housestaff used ultrasound guidance for most CVC placements [20].

Since in our department oncologists and hematologists have performed ultrasound imaging procedures as well as interventional ultrasound in clinical practice for patient management (diagnosis, staging, restaging and follow-up) for almost 25 years [21-27], this procedure was early applied as a guidance for CVC insertion in the internal jugular vein in cancer patients before the recommendations from the Agency for Healthcare Research and Quality were available [17,18]. Aims of this prospective observational study were to evaluate the safety and the efficacy of ultrasound-guided insertional CVC in patients with hematological and solid tumors, to confirm the utility of this procedure in clinical practice, and to evaluate local or systemic infection, CVC-related deep venous thrombosis (DVT) and lifespan of the CVC.

## Materials and methods

Adult patients with hematologic and solid tumors admitted to the oncology-hematology department of the hospital of Piacenza, North Italy, and requiring an indwelling CVC were offered the opportunity to partecipate in the study. There were 802 women (48%) and 858 men (52%) with a mean age of 61.72 years (range 18-85).

No exclusion criteria were contemplated except patients' refusal and patients candidate to palliative care only. The study was approved by the local ethics committee as a prospective observational study, and all the patients gave informed written consent before enrolment. Three types of CVC were employed according to different indications: single-lumen 16 gauge and, double-lumen Becton Dickinson (Singapore), double-lumen 13 French Arrow International (Erding, Germany). The last one was specifically employed in leukapheresis procedures. Each CVC-positioning is considered a single procedure for the study; so a patient who need catheter insertion more than once through his clinical history was registered as a new procedure every time CVC was inserted again.

The indications of CVC were: chemotherapy delivery, transfusion, parenteral nutrition, leukapheresis, autologous and allogenic stem cell transplantation, invasive hemodynamic variables assessment and blood sampling. Patients candidate to palliative care only were excluded from this study since they were enrolled in a different study.

Operators included two physicians and a nurse with specific experience with ultrasound and ultrasonically guided procedures, so that the level of experience of the operators would not bias results.

Patients were postured in the Trendelenburg's position with the head rotated toward the opposite side. The CVC was routinely implanted on the right side; however, if conditions were unsuitable for implantation on this side, such as in the case of lymphoadenopathy, or postradiation therapy area, or at the patient's request, the CVC was placed on the left side. All procedures were performed using standard aseptic techniques and a local anesthesia with a very small, 22-gauge needle for the venipuncture was applied under ultrasound guidance.

The ultrasound examinations were performed using ESAOTE (Genova, Italy) equipped with two transducers between 3.5 to 7.5 MHZ, with a needle guide, without a sterile cover. The method that we commonly use is "the three-handed method" [28]; this method requires an assistant to hold the probe, while the operator controls the needle and performs the procedure under real-time guidance, and the nurse helps the two physicians during the maneuver.

The central vein was identified along its greater longitudinal axis and its relationship with other anatomical structures using Valsalva's maneuver which determines an increase of the diameter of the veins. Under ultrasound-guide in real time, a 16-gauge needle is introduced into the last portion of internal jugular vein. This vein was reached through the transducer placed at the point of insertion of the sternocleidomastoid muscle into the clavicular; the correct introduction of the needle was always confirmed by ultrasound guidance and by the easy aspiration of venous blood.

The Seldinger technique was used to place the catheter, which was advanced into the superior vena cava until insertion into right atrium.

Every procedure was scheduled in order to register patient's data, pathological diagnosis, indications for CVC insertion, type of CVC, number of attempts and early complications if any failure; the procedures were observed by an independent team. Medications, CVC-related blood stream infection, symptomatic deep-vein thrombosis and CVC removal or substitution were also recorded. Within two hours after each procedure, chest radiography and ultrasound scanning were carried out to exclude pneumotorax and to evaluate correct catheter position. At the end of treatment or when required, after the removal of the catheter, the tip was sent to the laboratory for bacteriological examination.

No systemic prophylaxis against deep-vein thrombosis was adopted and no antibiotic prophylaxis was made. Each catheter at the end of its routine use was flushed with 20 ml normal sterile saline, then 5 ml heparinized saline (50 IU/ml). Follow-ups for each patient were scheduled every 10 days during the first six months, or until removal of the CVC. The follow-up consisted of: patient's clinical examination, catheters flushed with heparinized solution and catheter exit side dressing.

### Assesment of endpoints

The primary endpoints were: number of pneumotorax, accidental arterial and nerve puncture; major bleeding; number of attempts; failure; local hematoma.

Secondary endpoints were: symptomatic vein thrombosis of upper limbs (early or late), infections, malfunctioning and lifespan of the CVC.

In cases of clinical suspicion of venous thrombosis evidenced by progressive arm or facial swelling, the ultrasound criteria we considered in order to show the presence of catheter-related thrombosis included visualization of thrombus, absence of spontaneous flow, dilatation of the vein by the Valsalva maneuver and compressibility of the jugular vein as previously reported [29,30]. Infections were defined as catheter-related bacteremia: isolation of the same organism from the catheter in the presence of more than 15 colony-forming-units (CFUs) and positive blood culture without clinical signs of infection and catheter-related septicemia: isolation of the same organism from the catheter in the presence of more than 15 CFUs and positive blood culture, with clinical signs of infection [29]. Infections with a clinically apparent focus other than exit site or catheter were excluded. Catheter malfunctioning occlusion was registered when presented.

### Statistical analysis

Demographic data and clinical features were analyzed using descriptive methods. Quantitative variables were summarized using mean and standard deviation. Categorical variables were summarized as counts and percentages. Baseline analysis included all enrolled patients. Statistical tests were performed with Statview Software, latest version.

## Results

From 2 December 2000 to 31 January 2009, 1,978 CVC insertional procedures were applied in 1,660 patients. The procedure was performed 580 times in hematologic malignancies and 1,398 times in solid tumors (Table 1); the majority of patients with solid tumors had gastrointestinal cancer and the majority of patients with hematologic malignancies had lymphomas (Table 2). The median platelet count at the time of CVC insertion was 236,000/mm3 while the median neutrophil count was 4,015/mm3 (Table 3). It must be emphasized that a subset of patients, especially hematological ones, had neutropenia and low platelet count; neutropenia: 254 patients (15.3%) with neutrophils below 1,500/mm3, 132 (7.95%) below 500/mm3, 88 (5.3%) below 100/mm3; low platelet count: 116 patients (6.99%) with platelet count below 50,000/mm3, 70 (4.22%) below 20,000/mm3 and 38 (2.29%) below 10,000/mm3 (Table 4).

**Table 1:** Patients characteristics

	**N**^**o **^**of patients**	% of patients
Total	1,660	100
Median age	61.71 years (range 18-85)	
Male	858	52
Female	802	48
Type of cancer		
Solid tumor	1,280	77
Hematologic malignancies	380	23

**Table 2:** Types of cancer

	**N**^**o **^**of procedures**	% of cancer
Total	1,978	100

**Solid tumor**	**1,398**	70.68

Gastrointestinal	904	45.70

Lung	64	3.24

Breast	136	6.88

Other solid cancer	294	14.86

**Hematologic malignancies**	**580**	29.32

Lymphomas	216	10.82

Acute leukemia	196	9.91

Multiple myeloma	126	6.37

Other hematologic malignancies	42	2.12

**Table 3:** Main Hematologic characteristics of the overall population

	Median	Range	Normal range
Platelet count N/mm3	236,000	7,000-510,000/mm3	130,000-450,000/mm3

Neutrophil count N/mm3	4,015	85-10,500/mm3	

Prothrombin activity (%)	60	30-100	70-120 (%)

Fibrinogen (mg/dl)	185	100-460	150-400 (mg/dl)

APTT (sec)	30	25-45	26.50-37.50 (sec)

**Table 4:** Patients with impaired neutrophil/platelet count who underwent ultrasound guided internal jugular vein catheterization

Patients with neutropenia 254 of 1660			Patients with low platelet count 116 of 1660		
**N. Patients**	**% of patients**	**N. neutrophil/mm3**	**N. Patients**	**% of patients**	**N. Platelet/mm3**

254	15.3%	<1,500	116	6.99	<50

132	7.95	<500	70	4.22	<20

88	5.3	<100	38	2.29	<10

Primary end points: a single-needle puncture of the vein was performed on 1,948 of 1,978 procedures (98.48%) and eighteen attempts of the 1,978 failed (0.9%), so the procedure revealed itself to be efficacious in 99.1% of cases. Failures were caused by arterial puncture in 6 (0.3%), mediastinum CVC dislocation in 2 (0.1%), vein collapse in 8 (0.48%) and no efficacious "eco window" in 2 (0.1%). There were no differences in mechanical complications, malposition and malfunctioning of CVC with the right (94.7% of cases) and left side (5.3% of cases) approaches.

No major bleeding and no nerve puncture were reported; four (0.2%) self-limiting hematomas at the site of CVC insertion without requiring any medical intervention were registered after arterial puncture. No pneumotorax was reported (Table 5).

**Table 5:** Results of ultrasound guided catheter insertion, primary endpoints and mechanical complications

	**N**^**o**^	%
**Total procedures**	1,978	100
Access with one attempt	1,948	98.48
Access with two attempts	12	0,6
Pneumothorax	0	0
Major bleeding	0	0
Arterial puncture	6	0.3
Failure	18	0.9
Local Hematoma	4	0.2
Nerve puncture	0	0

### Secondary end points

Catheter-related infection occurred in 197/1,978 (9.96%) of the catheters inserted; CVC-related septicemia was observed in 98 cases (4.95%). No death was attributable to catheter-related infections (Table 6).

**Table 6:** Secondary endpoints and non-mechanical complications of ultrasound guided catheter insertion

	N	%
Total procedures	1,978	100
CVC-related infection	197	9.96
Septicemia	98	4.95
CVC-related symptomatic vein thrombosis	45	2.28
CVC removal due infection/thrombosis or malfunctioning	48	2.4
**Mean lifespan of CVC and standard devation**	**189.7, days +/- 18.6(range 7-701 days)**	

The organisms that caused catheter colonization were coagulase negative staphylococci 82%, Escherichia Coli 8%, Pseudomonas 6%, Enterococcus 4%.

The mean lifespan of CVC was 189.7 +/- 18.6 days (range 7-701), and CVC inserted under US guidance permitted the delivery of the planned therapy in 1,930/1,978 (97.6%) of patients.

In our 197 patients with catheter - related infection, antibiotic therapy resolved the infection: in 129 cases (65.5%) with CVC substitution and 20 cases (10.10%) without it. Only in 48 cases (24.40%) was definitive CVC removal required for associated thrombosis and for antibiotic therapy failure or for CVC malfunctioning.

Symptomatic deep-vein thrombosis of upper limbs developed in 45 cases (2.7%) mainly in the first 30 days of implantation. Clinical diagnosis suggested by progressive arm or facial swelling was confirmed by echodoppler. In 30/45 (66.7%) cases with thrombosis, CVC was removed because of associated infective complications (Table 6). No patient had any further thromboembolic complications. There was no difference in infection, bleeding or thrombosis in patients with single-lumen and double-lumen catheters; however the large - bore catheter employed in leukapheresis was generally removed after the procedure.

## Discussion

The use of central venous access devices has become an essential component of the treatment of many medical disorders. Central venous access is commonly attempted in the internal jugular vein, subclavian vein, femoral vein, or arm veins using peripherally central catheters. It is estimated that several million devices are inserted each year, facilitating many emerging therapies, including long-term chemotherapy [30].

Central venous cannulation can be unsafe: the National Confidential Enquiry into perioperative deaths has reported one death resulting from a procedure-induced pneumothorax [31].

It must be emphasized that less serious discomfort (though unpleasant for the patient), clinician time, hospital stay and economic costs are variable rates for failure and complications from central venous cannulation. Ten of 328 oncological patients (3.4%) developed pneumothorax after central venous access implanted without US guidance and 6 of them (60%) required tube-thoracostomies [30].

More recently, the etiology and incidence of iatrogenic pneumothorax, which can develop after invasive procedures performed for diagnostic and for therapeutic purposes, has been reported [32]: the most frequent procedure type causing pneumotorax was central venous catheterization, with 72 patients (43.8%) of the series of 164 patients developing iatrogenic pneumothorax.

In addition, complications are more frequent when more needle passes are necessary because of anatomical variation or difficult veins (small veins; no palpable landmarkers). Anatomical variations of the internal jugular vein were found in 8% of patients studied with ultrasound-guided CVC [33]; the ease and the shorter time required to perform ultrasound-guided catheterization, together with the higher rate of success and decreased incidence of complications make using the ultrasound-guided CVC preferable to conventional CVC [34-36].

In this large series of 1,978 CVC insertional performed procedures in 1,660 consecutive patients suffering from cancer in different phases of their disease, none of the patients experienced major complications (pneumotorax, hemothorax, nerve lesions). To our knowledge, this is the largest series of consecutive CVC insertional procedures performed in patients with hematologic malignancies and solid tumors and we have shown that this technique may be efficacious and safe both in patients with acute leukemia and with solid tumors. In addition, we emphasize that the catheter was inserted in 254 patients (15.3%) with neutrophils <1,500 ul and 116 patients (6.99%) with platelets <50 ul without complications. Individual institution data might differ from those gathered at other centers; however we think the results of this study are particularly relevant because they have been obtained through a large series of patients investigated with an accurate prospective trial performed over a long period of time. Our results can be favourably compared with a recent report where adverse events occurred in 26 cases (5.2%) of 500 US-guided CVC via subclavian vein, with two cases of pneumothorax [37].

In our study, the rate of CVC-related deep-vein thrombosis was low despite the absence of antithrombotic drugs in the practice of prophylaxis against venous thromboembolism in the patients enrolled; the incidence of symptomatic DVT was quite similar, although a bit low, with respect to the value in the control arm of previous studies which compared no systemic prophylaxis versus deltaparin or low-dose warfarin [38-40]. The low incidence of upper-limb DVT may be explained by ultrasound guide as previously stated [41] which allowed us to achieve the following targets:

1) to identify the exact site of CVC insertion, 2) to perform only one attempt in nearly all the patients (98.48%), causing little damage to the venous endothelium: in fact, it is well known that endothelium damage is a main risk factor in initiating thrombosis.

However the lack of venographic assessment of catheter-related thrombosis in our study is a limit to the determination of the real frequency of this complication, as previously reported [40].

The incidence of CVC infective complication was similar to previous data concerning non-tunnelled catheters, while it was higher than totally implantable central venous access ports [29,42]. The great incidence of staphylococci reflects the classical expositional risk of this kind of catheter, although the incidence of infections, the rate of CVC withdrawal or malfunction was low when compared with previous reports [29], since prompt antibiotic therapy allowed a good control of bacterial infection. Moreover, our non-tunnelled catheters were easily replaced whenever clinical conditions suggested it. In the present study, ultrasound-guided CVC procedures were performed by our own specifically experienced oncologists and hematologists, and we agree with a recent report [28] that ultrasound is an easily learned technique that not only enhances the physical examination, but has the distinct advantage of being a portable tool that can provide real-time guidance for CVC insertion and other interventional procedures such as biopsy, abscess drainage, paracentesis, thoracentesis, etc, and for critically ill and elderly patients the procedure can be performed easily at the bedside as previously reported [21-27]. Physicians with specific experience were defined as operators who have performed > 50 CVC insertion under ultrasound guidance [4,28].

It must be emphasized that the use of ultrasound is not limited to radiologists. The American Medical Association policy fovours ultrasound imaging technique diffusion in medical practice [43], the American College of Emergency Physicians and the American College of Surgeons support the use of ultrasound by members of their societies and address ways to obtain and maintain competence as well as ensuring quality control [44,45].

We believe that this technique is very useful also in the oncology and hematology department, not only for CVC placement but also for staging, disease control, follow-up and ultrasound guided-procedures [21-27].

## Conclusion

Our large study show that ultrasound-guided CVC insertion is a safe and effective technique that is sufficient to give a patient six or more months of treatment. Safety and efficacy of the procedure did not negatively impact on infective and thrombotic complications. We believe this technique can be applied easily to most practitioners in clinical oncology with relevant patient benefits.

## Competing interests

The authors declare that they have no competing interests.

## Authors' contributions

Study Concepts: LC, GC, DV. Study Design: LC, GC, DV. Data Acquisition: From LC to LT. Quality Control of Data and Algorithms: LC, GC, DV and CB. Data Analysis and Interpretation: CB and LC. Statistical analysis: DV and CB. Manuscript preparation: LC, CB, CDN. Manuscript editing: LC, CB, CDN. Manuscript review: all authors.

## References

[B1] McGee DC Gould MK Current concepts: preventing complications of central venous catheterisation N Engl J Med 2003 348 1123 1133 10.1056/NEJMra011883 12646670

[B2] Cortelezzi A Fracchiola NS Maisonneuve P Moia M Luchesini C Ranzi ML Monni P Pasquini MC Lambertenghi-Deliliers G Central venous catheter-related complications in patients with hematological malignancies. A retrospective analysis of risk factors and prophylactic measures Leu Lymph 2003 44 1495 1501 10.1080/1042819031000103980 14565650

[B3] Bernard RW Sthal WM Subclavian vein catheterizations: a prospective study: i. Non-infectious complications Ann Surg 1971 173 184 190 10.1097/00000658-197102000-00002 5100094 PMC1397638

[B4] Sznajder JI Zveibil FR Bitterman H Weiner P Bursztein S Central vein catheterization: failure and complication rates by three percutaneous approaches Arch Intern Med 1986 146 259 261 10.1001/archinte.146.2.259 3947185

[B5] Defalque RJ Percutaneous catheterizations of the internal jugular vein Anesth Analg 1974 53 116 121 4589503

[B6] Mallory DL McGee WT Shawker TH Brenner M Bailey KR Evans RG Parker MM Farmer JC Parillo JE Ultrasound guidance improves the success rate of internal jugular vein cannulation: a prospective, randomized trial Chest 1990 98 157 160 10.1378/chest.98.1.157 2193776

[B7] Troianos CA Jobes DR Ellison N Ultrasound-guided can nulation of the internal jugular vein: a prospective, randomized study Anesth Analg 1991 72 823 826 10.1213/00000539-199106000-00020 2035868

[B8] Denys BG Uretsky BF Reddy PS Ultrasound-assisted cannulation of the internal jugular vein: a prospective comparison to the external landmark-guided technique Circulation 1993 87 1557 1562 8491011 10.1161/01.cir.87.5.1557

[B9] Slama M Novara A Safavian A Ossart M Safar M Fagon JY Improvement of internal jugular vein cannulation using an ultrasound-guided technique Intensive Care Med 1997 23 916 919 10.1007/s001340050432 9310813

[B10] Teichgraber UK Benter T Gebel M Manns MP A sonographically guided technique for central venous access AJR Am J Roentgenol 1997 169 731 733 9275887 10.2214/ajr.169.3.9275887

[B11] Nadig C Leidig M Schmiedeke T Höffken B The use of ultrasound for the placement of dialysis catheters Nephrol Dial Transplant 1998 13 978 981 10.1093/ndt/13.4.978 9568861

[B12] Hayashi H Amano M Does ultrasound imaging before puncture facilitate internal jugular vein cannulation? Prospective randomized comparison with landmark-guided puncture in ventilated patients J Cardiothorac Vasc Anesth 2002 16 572 575 10.1053/jcan.2002.126950 12407608

[B13] Leung J Duffy M Finckh A Real-time ultrasonographicallyguided internal jugular vein catheterization in the emergency department increases success rates and reduces complica tions: a randomized, prospective study Ann Emerg Med 2006 48 540 547 10.1016/j.annemergmed.2006.01.011 17052555

[B14] Karakitsos D Labropoulos N De Groot E Patrianakos AP Kouraklis G Poularas J Samonis G Tsoutsos DA Konstadoulakis MM Karabinis A Real-time ultrasound guided catheterization of the internal jugular vein: a prospective comparison to the landmark technique in critical care patients Crit Care 2006 10 R162 10.1186/cc5101 17112371 PMC1794469

[B15] Randolph AG Cook DJ Gonzales CA Pribble CG Ultrasound guidance for placement of central venous catheters: a meta-analysis of the literature Crit Care Med 1996 24 2053 2058 10.1097/00003246-199612000-00020 8968276

[B16] Hind D Calvert N McWilliams R Davidson A Paisley S Beverley C Thomas S Ultrasonic locating devices for central venous cannulation: meta-analysis BMJ 2003 327 361 10.1136/bmj.327.7411.361 12919984 PMC175809

[B17] Rothshild JM Ultrasound guidance of central vein catheterization 2001 http://archive.ahrq.gov/clinic/ptsafety/chap21.htm Accessed June 4, 200711524913

[B18] Making health care safer: a critical analysis of patient safety practices 2001 http://www.ahrq.gov/clinic/ptsafety/Accessed June 4, 2007 PMC4781305 11510252

[B19] Howard S A survey measuring the impact of NICE guidance 49: the use of ultrasound locating devices for placing central venous catheters 2004 http://www.nice.org.uk/nicemedia/live/11474/32466/32466.pdf

[B20] Girard TD Schectman JM Ultrasound guidance during central venous catheterization: a survey of use by house staff physicians J Crit Care 2005 20 224 229 10.1016/j.jcrc.2005.06.005 16253790

[B21] Cavanna L Di Stasi M Fornari F Civardi G Sbolli G Buscarini E Buscarini L Ultrasound and ultrasonically guided biopsy in hepatic lymphoma Eur J Cancer 1987 23 3 323 6 10.1016/0277-5379(87)90076-9 3297720

[B22] Fornari F Civardi G Cavanna L Di Stasi M Rossi S Sbolli G Buscarini L Complications of ultrasonically guided fine-needle abdominal biopsy. Results of a multicenter Italian study and review of the literature. The Cooperative Italian Study Group Scand J Gastroenterol 1989 24 8 949 55 10.3109/00365528909089239 2688068

[B23] Sbolli G Fornari F Civardi G Di Stasi M Cavanna L Buscarini E Buscarini L Role of ultrasound guided fine needle aspiration biopsy in the diagnosis of hepatocellular carcinoma Gut 1990 31 11 1303 5 10.1136/gut.31.11.1303 2174818 PMC1378704

[B24] Cavanna L Civardi G Fornari F Di Stasi M Sbolli G Buscarini E Vallisa D Rossi S Tansini P Buscarini L Ultrasonically guided percutaneous splenic tissue core biopsy in patients with malignant lymphomas Cancer 1992 69 12 2932 6 15 10.1002/1097-0142(19920615)69:12<2932::AID-CNCR2820691211>3.0.CO;2-7 1591686

[B25] Civardi G Vallisa D Bertè R Giorgio A Filice C Caremani M Caturelli E Pompili M De Sio I Buscarini E Cavanna L Ultrasound-guided fine needle biopsy of the spleen; high clinical efficacy and low risk in a multicenter Italian study Am J Hematol 2001 67 2 93 9 10.1002/ajh.1085 11343380

[B26] Civardi G Vallisa D Berte' R Lazzaro A Moroni CF Cavanna L Focal liver lesions in non-Hodgkin's lymphoma: investigation of their prevalence, clinical significance and the role of Hepatitis C virus infection Eur J Cancer 2002 38 18 2382 7 10.1016/S0959-8049(02)00481-1 12460782

[B27] Cavanna L Lazzaro A Vallisa D Civardi G Artioli F Role of image-guided fine needle-aspiration biopsy in the management of patients with splenic metastasis Word J Surg Oncol 2007 2 5 13 10.1186/1477-7819-5-13 PMC1800304 17274814

[B28] Feller Kopman-David Ultrasound-guided Internal Jugular Access: a proposed standardized approach and implications for training and practice Chest 2007 132 302 9 10.1378/chest.06-2711 17625091

[B29] Harter C Salwender HJ Bach A Egerer G Goldschmidt H Ho AD Catheter-related infection and thrombosis of the internal jugular vein in hematologic-oncologic patients undergoing chemotherapy. A prospective comparison of silver-coated and uncoated catheters Cancer 2002 94 245 51 10.1002/cncr.10199 11815983

[B30] Biffi R De Braund R Orsi F Pozzi S Mauri S Goldhirsch A Nolè F Andreoni B Totally implantable central venous access ports long-term chemotherapy Annals of Oncology 1998 9 767 773 10.1023/A:1008392423469 9739444

[B31] Callum KG Whimster F International vascular radiology and neurovascular radiology: a report of the National Confidential Enquiry into Perioperative Deaths. Data collection period 1 April 1998 to 31 Mar 1999 2000 London, NCEPOD

[B32] Celik B Sahin E Nadir A Kaptanoglu M Iatrogenic pneumotorax etiology incidence and risk factors Thorac Cardiovasc Surg 2009 57 286 90 10.1055/s-0029-1185365 19629891

[B33] Danys BG Uretsky BF Reddy PS Ultrasound assisted cannulation of the internal jugular vein Crit Care Med 1991 87 1557 62 10.1161/01.cir.87.5.1557 8491011

[B34] Lameris JS Post PJM Zonderland HM Gerritsen PG Kappers-Klunne MC Schütte HE Percutaneous placement of Hickmann catheters: comparison of sonografically guided and blind techniques Am J Radiol 1990 155 1097 9 10.2214/ajr.155.5.2120941 2120941

[B35] Mallory DL Megee WT Ultrasound guidance improves the success rate of internal jugular vein cannulation Circulation 1993 87 1557 62 2193776 10.1378/chest.98.1.157

[B36] Cajozzo M Quintini G Cocchiera G Greco G Vaglica R Pezzano G Barbera V Modica G Comparison of central venous catheterization with and without ultrasound guide Transfusion and Apheresis Science 2004 31 199 202 10.1016/j.transci.2004.05.006 15556467

[B37] Sakamoto N Arai Y Takeuchi M Ultrasound - Guided Radiological Placement of Central Venous Port via the Subclavian Vein: A restrospective Analysis of 500 Cases at a Single Institute Cardiovasc Intervent Radiol 2010 33 989 94 10.1007/s00270-010-9841-y 20390274

[B38] Couban S Goodyear M Burnell M Dolan S Wasi P Barnes D Macleod D Burton E Andreou P Anderson DR Randomized placebo-controlled study of low-dose warfarin for the prevention of central venous catheter-associated thrombosis in patients with cancer J Clin Oncol 2005 23 4063 9 10.1200/JCO.2005.10.192 15767639

[B39] Karthaus M Kretzschmar A Kroning H Biakhov M Irwin D Marschner N Slabber C Fountzilas G Garin A Abecasis NG Baronius W Steger GG Südhoff T Giorgetti C Reichardt P Dalteparin for prevention of catheter-related complications in cancer patients with central venous catheters: final results of a double-blind, placebo-controlled phase III trial Ann Oncol 2005 17 289 96 10.1093/annonc/mdj059 16317012

[B40] Fagnani D Franchi R Porta C Pugliese P Borgonovo K Bertolini A Duro M Ardizzoia A Filipazzi V Isa L Vergani C Milani M Cimminiello C POLONORD Group Thrombosis-related complications and mortality in cancer patients with central venous devices: an observational study on the effect of antithrombotic prophylaxis Ann Oncol 2007 18 551 5 10.1093/annonc/mdl431 17158773

[B41] Verso M Agnelli G Venous thromboembolism associated with long-term use of central venous catheters in cancer patients J Clin Oncol 2003 21 3665 75 10.1200/JCO.2003.08.008 14512399

[B42] Gallieni M Pittiruti M Biffi R Vascular access in oncology patients A Cancer J Clin 2008 58 323 46 10.3322/CA.2008.0015 18971486

[B43] American Medical Association Res. 802, I-99 and Reaffirmed: Sub.Res. 108, A-00; privileging for ultrasound imaging 2005 http://www.acep.org/WorkArea/DownloadAsset.aspx?id=44304 Accessed March 15, 2006

[B44] American College of Emergency Physicians AECP policy state-ment: emergency ultrasound guidelines 2001 http://www.acep.org/practres.aspx?id=32334 Accessed June 4, 2007 11574810

[B45] American College of Surgeons Ultrasound examinations by surgeons 1998 http://www.facs.org/fellows_info/statements/statement.html Accessed June 4, 2007

